# The epigenetic regulation of crosstalk between cardiac fibroblasts and other cardiac cell types during stress

**DOI:** 10.3389/fcvm.2025.1539826

**Published:** 2025-04-08

**Authors:** Lindsay Kraus, Synclare Fredericks, Katelyn Scheeler

**Affiliations:** Department of Biology, College of Science, Technology, Engineering, Arts, and Mathematics, Alvernia University, Reading, PA, United States

**Keywords:** epigenetics, cardiovascular disease, cardiac fibroblast (CF), crosstalk, extracellular matrice (ECM)

## Abstract

With the global impact of cardiovascular disease, there is a dire need to understand the mechanisms in the heart during injury and stress. It has been shown that the regulation of the extracellular matrix via cardiac fibroblasts plays a major role in the progression of heart failure and worsening function of the heart. Importantly, it has been suggested that crosstalk between other cardiac cells like cardiomyocytes, immune cells, and endothelial cells are influenced by the pathological function of the fibroblasts. This decline in function across all cardiac cells is seemingly irreversible. However, epigenetic mechanisms have been shown to regulate functionality across cardiac cells and improve outcomes during stress or injury. This epigenetic regulation has also been shown to control communication between different cell types and influence the role of multiple cardiac cell types during injury. The goal of this review is to summarize and discuss the current research of epigenetic regulation of cardiac fibroblasts and the subsequent crosstalk with other cardiac cell types in cardiovascular disease states.

## Graphical Abstract

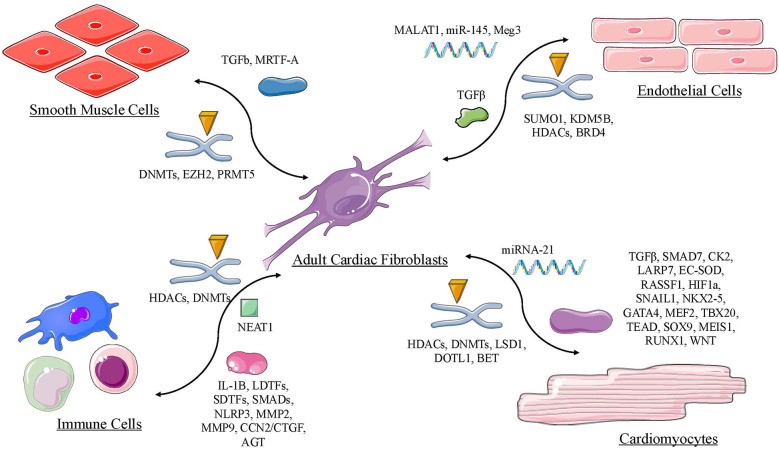

Graphical abstract summarizing the role and mechanisms of epigenetic regulation between cardiac fibroblasts and other cardiac cell types.

## 1 Introduction

Cardiovascular diseases (CVDs) are the leading causes of death worldwide, with over 17 million deaths annually (1, 2). There is a crucial need to lessen the global impact of CVDs, and with no cure currently, it is vital to gain understanding of the heart and its pathologies (3). To best understand cardiac biology, it is necessary to explore the regulation of the specific cell types within the heart and how they communicate with each other, particularly in times of stress, injury, or disease.

The heart is composed of many cell types including fibroblasts, cardiomyocytes, immune cells, and endothelial cells (4). In CVDs, these cells can be damaged, lost, or altered in a way that further exacerbates damage in the heart (5, 6). For example, it has been reported that as many as 25% of cardiomyocytes can be lost irreparably after a myocardial infarction (7). Cardiac fibroblasts can be lost as well; however, they are more often observed differentiating into a pathological phenotype which, if not properly regulated increases scarring and decreases overall heart function (8). These changes often become irreversible, leading to the inevitable decline of heart function (9).

It has been shown that cardiac fibroblasts (CFs) maintain an important balance in the heart in both healthy and pathological states. It is well understood that CFs regulate the cardiac environment via the extracellular matrix (ECM). The ECM is vital to maintain healthy heart function as well as managing the heart during stress or injury (10). CFs modify the ECM with proteins like collagens and growth factors, as well as through genetic and epigenetic modifications (11–13).

Many studies explore the isolated role of CFs or other cells commonly found in the heart, but research has shown that many cardiac cells are constantly communicating with each other via the ECM (14, 15). Often, this is described as crosstalk, and has been suggested as a pivotal mechanism for understanding cardiovascular therapies (16). Especially in injury associated with CVDs, this crosstalk is an important component to further understand scarring, inflammation, and wound healing (17). To date, research and reviews have indicated and studied this crosstalk led by CFs, but the regulation behind this communication is still not as well understood.

Recently, research has shown that epigenetic regulation plays a major role in crosstalk between CFs and other cardiac cells (18, 19). Epigenetics, or external modifications to the DNA, are known to occur throughout development and even during stress or injury. In general, these direct epigenetic regulators are deemed writers, readers, or erasers based on their ability to add, interpret, or remove histone modifications such as methylation or acetylation on histones or DNA (20, 21). Some of the most common changes of this type of gene regulation include DNA methylation and demethylation (DNMTs), histone methylation, acetylation or deacetylation (HMT, HATs and HDACs) and noncoding RNA interplay (microRNAs and long noncoding RNAs) (22). Particularly, epigenetic regulation of CFs has been well studied in many scenarios, including CVDs, aging, immune regulation, and cancer (23, 24). More specifically, the direct effect of epigenetic changes has been shown to alter crosstalk between CFs and myocytes, endothelial cells, and immune cells (19). Epigenetic modifications are not always dictated by direct modifiers, like histone proteins. Epigenetic regulation can be modified via indirect mechanisms, like small RNA molecules, signal proteins, and reactive oxygen species. Withmore understanding of epigenetic regulation, there is an intertwining relationship between epigenetic and genetic influence on cell phenotype. The goal of this review is to summarize the current role of all of these various epigenetic mechanimsof crosstalk between cardiac fibroblasts and the most common cardiac cells found in the heart as shown in Table 1. This will providea novel understanding of the pathological progression as well as the potential therapeutic approach for CVDs.

**Table 1:** Summary of key epigenetic regulation mechanisms involved in the crosstalk with cardiac fibroblasts and other cardiac cell types.

Cardiac Cell Crosstalk Interaction	Epigenetic mechanism, modification, or regulation	Source
Cardiac Fibroblasts and Cardiomyocytes	*Small molecules*	30, 31, 32, 36, 37, 39, 40, 41, 43, 45, 47
	- miR-21	
	*Signal proteins*	
	- TGFβ, SMAD7, CK2, LARP7, EC-SOD, RASSF1, HIF1*α*, SNAIL1, NKX2-5, GATA4, MEF2, TBX20, TEAD, SOX9, MEIS1, RUNX1, WNT	
	*Epigenetic regulators*	
	- HDACs, DNMTs, LSD1, DOTL1, BET	
Cardiac Fibroblasts and Smooth Muscle Cells	*Signal proteins*	52, 53, 54, 57, 59
	- TGFβ, MRTF-A	
	*Epigenetic regulators*	
	- DNMTs, EZH2, PRMT5	
Cardiac Fibroblasts and Immune Cells	*Small molecules*	63, 64, 67, 68, 69, 71
	- NEAT1	
	*Signal proteins*	
	- IL-1B, LDTFs, SDTFs, SMADs, NLRP3, MMP2, MMP9, CCN2/CTGF, AGT	
	*Epigenetic regulators*	
	- HDACs, DNMTs,	
Cardiac Fibroblasts and Endothelial Cells	*Genome editing*	73, 74, 78, 79, 83, 84, 85
	- CRISPRa-SAM	
	*Small molecules*	
	- MALAT1, miR-145, Meg3	
	*Signal proteins*	
	- TGFβ,	
	*Epigenetic regulators*	
	- SUMO1, KDM5B, HDACs, BRD4	

## 2 Epigenetic regulation cardiac fibroblast with other cardiac cell types

### 2.1 Epigenetic regulation between cardiac fibroblasts and adult cardiomyocytes

When looking at adult myocardium, cardiomyocytes (CMs) are one of the most important cell types to understanding heart function. Research has suggested that CMs account for about 64% of the left ventricle and about 37% of the right ventricle (4); they account for 60%–70% of the heart volume (25). They are the contractile cells of the myocardium that, under normal conditions, contract and relax the heart (26). The intercellular connection between CMs and CFs has been a heavily studied topic to understand how the heart responds to pathogenic stimuli. It is well known that these two cell types communicate to each other directly through tunneling nanotubes and gap junctions as well as indirectly via signaling pathways, paracrine molecules, and through the ECM (27–29). The transcriptional activity of a cell involves both epigenetic factors and genetic material. This section will highlight which epigenetic modifications are present in CM-CF crosstalk and how certain molecules and signaling proteins are involved in epigenetic regulation. Focusing on the underlying epigenetic mechanisms will allow us to better understand the bidirectional crosstalk in the adult human heart.

Small RNA molecules such as microRNA (miRNA) strongly influence the communication between CFs and CMs. A study by Pradhan et al. showed that individuals with a history of atrial fibrillation (AF) had a high expression level of microRNA-21-5p (miR-21). This non-coding RNA is a regulatory molecule responsible for modifying different elements of the epigenome. MiR-21 is released from CMs in response to stress stimuli which induces fibrotic effects by the promotion of excessive collagen production in CFs (30). Medium from paced cardiomyocytes were transferred to cultured fibroblasts. After a fibrosis profiling array, many upregulated profibrotic transcripts were related to transforming growth factor beta (TGF-β). This cytokine is responsible for proliferation and collagen secretion in CFs, and myofibroblast activation (31). Abnormal regulation of the TGF-β signaling pathway leads to maladaptive cardiac physiology (31). The researchers also found that a specific histone deacetylase (HDAC) inhibitor, mocetinostat, can attenuate miR-21 release in AF cardiomyocytes by inhibiting miR-21 expression. As a result, anti-fibrotic effects were observed from restoration of the expression of TGF-β pathway inhibitors, suppressor of mothers against decapentaplegic (SMAD)7 and Decorin, along with repressing activators associated with fibrosis development (30).

Signaling proteins have a huge impact in epigenetic regulation of crosstalk mainly through the previously mentioned TGF-β pathway. The importance of TGF-β is exemplified in a study by Basma et al. involving exosome signaling between CMs and CFs. Exosomes are extracellular vesicles that transport molecules such as proteins, metabolites, and lipids, facilitating cell to cell communication. CFs and their exosomes treated with TGF-β induced significant transcriptional changes resembling heart failure phenotype. Additionally, when co-cultured with CMs, TGF-β stimulated exosomes produced increased contraction and hypertrophic expression (32). When looking at the genes expressed in co-cultured cells, there was a high level of DNA methyltransferase (DNMT)3A and DNMT3B which suggested that exosomes from CFs produced a hypermethylation signal in CMs (32). DNMTs are enzymes that add chemical methyl groups to DNA which ultimately inhibits transcriptional activity. Exosomes signaling through CFs can serve as an important biomarker for discovering pathogenic conditions of the heart. Similarly, Cartledge et al. provided further evidence of the role of epigenetic regulation associated with paracrine signaling in CFs and CMs through soluble mediators. Co-culturing CFs with CMs and myofibroblasts showed hypertrophic effects, specifically altering CM viability, volume, and Ca2 + transients (33). The soluble mediators produced by CMs drove CF proliferation. Also, it was described that increased TGF-β secretion from CFs was by cause of co-culturing with CMs. TGF-β proved to be the cause of the hypertrophic expression (33). Reduction of viability and volume in CMs was prevented by blocking the TGF-β type 1 receptor with the small molecule inhibitor SB431542 (33). This chemical compound is potent for inhibiting the activity of TGF-β across various cells and organ systems (34, 35).

The significance of TGF-β paracrine signaling extends to a study by Claridge et al. revealing the importance of the secretome in crosstalk between CMs and CFs. The CFs were treated with TGF-β-stimulated CMs, from human induced pluripotent stem cells, allowing them to assess the proteomic landscape and secretome-mediated signaling processes in those same fibroblasts (36). Disruption to their oxidative state and hyperactivation leading to a myofibroblast phenotype was likely due to the downregulation of antioxidant and reactive oxygen species (ROS)-related proteins. This pathological remodeling of the CFs created an unbalance in the proteomic landscape, through the ECM, leading to fibrosis effects. Some of the dysregulated components in the TGF-β-CM treated CFs included regulators of fibrosis such as angiotensin II receptor-associated protein, insulin-like growth factor 2, and prostaglandin F2 receptor negative regulator. Other transcriptional factors and epigenetic regulators with alterations included C-terminal binding protein 2, aquarius intron-binding spliceosomal factor, and HDAC2 (36). This suggests that TGF-β stimulation creates disruption in the signaling components and epigenetic modifiers from CMs to CFs.

Over time, oxidative stress has been linked to many different types of CVDs due to the harmful effects of ROS on cellular function. One study points out how ROS is involved in the altered expression of [La ribonucleoprotein 7] (LARP7) and how it can lead to heart failure physiology. Ribonucleoproteins regulate RNA processing and are involved in epigenetic regulation. Ataxia-telangiectasia mutated (ATM) modulates LARP7 expression in diseased neonatal and adult CMs (37). Oxidative stress caused by ROS can lead to ATM activation triggering the degradation of LARP7. ATM activation has also been found in fibroblasts (38). This leads to altered expression of proteins of an HDAC, Sirtuin 1 (SIRT1), further impairing mitochondrial biogenesis and functionality in CMs. Collagen genes that were abnormally expressed included type 1 collagen alpha 2 chain (Col1a2), Col4a1, Col5a2, Col6a (37). Using inhibitors of ATM, such as KU60019, ultimately restored levels of LARP7, SIRT1 and mitochondrial biogenesis preventing adverse remodeling effects of myocardial infarction (MI). Additionally, SRT1720 (SIRT1 activator) can improve mitochondrial function and attenuate hypertrophy and fibrosis of the heart. Adeno-associated virus gene therapy was shown to be a novel gateway into remediating the expression of LARP7. The ROS-ATM-LARP7-SIRT1 axis should be further studied to combat ROS induced cardiac dysfunction.

Continuing with the crucial impact of ROS, scientists found that overexpression of an antioxidant enzyme, extracellular superoxide dismutase (EC-SOD), ameliorates cardiac fibrosis and oxidative stress. Transgenic mice (TG) were given an extra copy of EC-SOD and put in hypoxic conditions. In the same conditions, control wildtype mice (WT) showed increased protein levels of Collagen 1, alpha smooth muscle actin (α-SMA), and zinc-finger protein (SNAIL1) parallel to higher levels of ROS and hypoxia-inducible transcription factor 1 α (HIF1α). The TG group revealed a reduction of Col1, Col3, α-SMA, ROS and HIF1α. Additionally, the methylation level of DNMT1 and DNMT3B were significantly lower in the TG group. These DNMTs were found to bind to promoter region of the tumor suppressing gene, Ras association domain family member 1 (RASSF1), inhibiting its expression (39). RASSF1 has been shown before to be an important factor in paracrine mediated signaling between CFs and CMs (40). In CFs, RASSF1 represses the transcriptional activity of nuclear factor kappa B and inhibits the production and secretion of tumor necrosis factor alpha. As a result, hypertrophic signaling between CFs and CMs is prevented. Chronic hypoxic stress can lead to methylation of RASSF1, subsequently activating the extracellular-signal regulated kinase 1 and 2 (ERK1/2) pathway leading to CF activation and proliferation (39). This study provides a novel mechanism by which EC-SOD can epigenetically regulate RASSF1 and further downstream signaling in CFs and CMs by abrogating DNA methylation.

A major factor contributing to cellular crosstalk between CFs and CMs are transcription factors (TF) and the enhancers they bind to. A study by Golan-Lagziel et al. highlighted critical CF/CM-specific cis-regulatory elements (CRE) such as enhancers and TFs to regulate gene expression. Using epigenomic sequencing techniques, they identified histone marks such as the acetylation of histone 3 (H3K27) which correlated to specific CREs in both CFs and CMs, increasing gene expression. The regulation of the transcriptional activity in these two cell types are coordinated by CREs and their respective TFs which happen to be clustered together combinatorically (41). In CMs they were able to identify homeobox proteins (NKX-2-5 and MEIS2), GATA, Myocyte enhancer factor 2, estrogen-related receptors, and TBOX 5/20 as the major TFs that bind to CM enhancers. In CFs, the major TFs that bind to the CF enhancers are Tea domain, SRY-box 9, SMAD, T-cell factor, MEIS1, Recombination signal binding protein for immunoglobulin kappa J, and Runt-related 1. Though the TFs were not identical in each cell type it did not take away from the fact that due to their combinatorial organization with cell-specific enhancers, they have a major impact on gene expression within CMs or CFs respectively. This suggests looking further into these transcription factors and enhancers they bind to and how they behave in response to epigenetic modifications in certain cardiac states of health and pathology.

The epigenetic landscape between these two cell types is not solely dependent on signaling proteins. There is evidence suggesting that some mitochondrial metabolic substrates play a role in modifying the epigenetic and transcriptomic landscape of fibroblasts and induced cardiomyocytes (iCMs) during direct cardiac conversion. The efficiency of the converting fibroblasts into iCMs has been shown to be influenced by mitophagy and clearance of damaged mitochondria, disengagement of anabolic pathways, tricarboxylic acid cycle dependency, and change in chromatin state through nutritional manipulation (42). Interestingly, low-lipid growth medium allowed mouse embryonic fibroblasts to increase the expression of cardiac muscle troponin T, a key gene expressed in CMs. Additionally, these iCMs generated in low-lipid conditions had noticeably more histone residues of dimethylation of histone 3 (H3K27me2) and unmodified H3K4 resembling a CM. By understanding the metabolic modulations that affect the environment of the epigenome and transcriptome, we can design more efficient methods of cardiac cell reprogramming to alleviate CVDs.

Beyond small molecules and proteins, direct epigenetic modifiers have a pivotal role in precisely orchestrating the crosstalk between these two cell types. Cell-specific loss of histone lysine demethylase 1, LSD1, in CFs and CMs regulates cardiac remodeling in distinct ways. Using short interfering RNA or a small molecule inhibitor ORY-1001, LSD1 can be knocked out for further evaluation in each cell type. Deficiency of LSD1 in CFs serves as a protective role of transverse aortic constriction-induced heart failure by inhibiting the TGF-β activation and phosphorylation of its downstream targets Smad2/3, tumor protein 38, ERK, and c-Jun N-terminal kinases (43). Other antifibrotic effects of LSD1 deletion in CFs resulted in downregulation of α-SMA, Col1a1, Col3a1, and Col11a genes. Co-culture experiments with CFs and CMs showed that LSD1 serves as a target for the crosstalk between the two cells through TGF-β. In contrast, LSD1-specific knockout in isolated CMs caused mild hypertrophy and cardiac dysfunction through the downregulation of RE1-silencing transcription factor and its corepressor which is linked to the upregulation of atrial natriuretic peptide (ANP) and brain natriuretic peptide (43). It is vital we continue to study LSD1 specific cell-cell interactions to better understand its purpose in CVD.

Another class of epigenetic regulators are histone methyltransferases (HMT). By adding methyl groups to histone residues, HMTs can affect the accessibility of chromatin, further impacting gene expression. Certain expression levels of disruptor of telomeric silencing 1-like (DOTL1) plays a role in the cardiac fibrotic response. DOTL1 is an HMT responsible for methylating H3K79. Rat CFs were stimulated with either TGF-β or Angiotensin II (Ang II) and the expression level of DOTL1 was markedly higher than in control conditions (44). By knocking out DOTL1 with small molecule inhibitor EPZ5676, fibrotic marker genes such as Fibronectin, Col3, Matrix metalloproteinase 9 and Connective tissue growth factor were all downregulated. This inhibitor can specifically decrease the trimethylation of H3K79which is associated with transcriptional activation of Forkhead box O 3a. Inhibition of DOTL1 also alleviates the cardiac remodeling response after induced MI.

A more novel layer of epigenetic regulation of crosstalk between CFs and CMs are Bromodomain and extraterminal (BET) proteins. BETs are acetyl-lysine interpreter proteins that play a major role in pathological cardiac remodeling. It is known that bromodomain (BRD)2/3/4 mRNA transcripts are found in neonatal and adult rat CFs and CMs. Of this class of proteins, BRD4 expression was elevated in CMs when given phenylephrine to induce hypertrophic effect (45). BRD4 signaling also caused the upregulation of pro-hypertrophic genes such as ANP. BET inhibitors, such as thienotriazolodiazepine (JQ1), can suppress these effects and signaling responses. In a similar study BET proteins were shown to induce proinflammatory and TGF-β gene networks in CFs and CMs which can result in heart failure (46). The application of JQ1 was also associated with attenuation of hypertrophic and fibrotic effects within the heart. Continuing to study the regulation of other BRD4 and other BET proteins will increase our understanding of the complex communication happening within these two cell types.

The epigenetic regulation of crosstalk between cardiac fibroblasts and neonatal cardiomyocytes is crucial to the development of the postnatal heart as well as the changing function of the heart during development. The relationship between fibroblasts and neonatal cardiomyocytes is still not fully understood, however, recent research has been able to determine some connections due to epigenetic regulation. Shortly after birth, the roles of fibroblast and neonatal cardiomyocytes quickly change. Neonatal cardiomyocytes will binucleate, stopping the cell cycle with the take up of hypertrophic growth. At the same time fibroblasts undergo rapid growth and multiplication producing an ECM which allows for the maturation of other cells through the creation of a fibrous backbone via the ECM (47). These changes in the components of the ECM from prenatal to postnatal environments provides the infrastructure for the crosstalk and epigenetic regulation of fibroblasts and neonatal cardiomyocytes. The internal environment of the heart changes rapidly within the first week post-birth. The amounts of cardiomyocytes and fibroblasts, their level of maturity, and their behavior can fluctuate within this time period. In the neonatal stage the heart has been shown to have self-restorative properties after injury. One way the heart can have these properties is through non-canonical Wnt signaling between neonatal cardiomyocytes and fibroblasts (47). In a research study investigating the restorative properties of the neonatal heart, it was also found that the changes leading to the maturation of the cells postnatal may be responsible for the loss of self-restorative properties the neonatal heart has (48).

It has been hypothesized that the main reason fibroblasts and neonatal cardiomyocytes are able to be epigenetically regulated is through the ECM and its components (47). As the components of the ECM change from the prenatal to postnatal environment, cardiomyocytes and fibroblast function also change. In the fetal heart the ECM is composed of fibronectin, immature collagen, and proteoglycans whereas after birth it is composed of FN, proteoglycans, an increase in fibrillar Col1 and Col3, laminin, LOX, Periostin, and a decrease in hyaluronic acid (47). The vastly different components of the ECM in different stages of the development of the heart are obvious. Many of the specific pathways for crosstalk between fibroblasts and neonatal cardiomyocytes are still unknown.

### 2.2 Epigenetic regulation between cardiac fibroblasts and smooth muscle cells

Another vital cardiac cell type, the smooth muscle cell (SMC), has been shown to be regulated by various epigenetic mechanisms. It has been documented that SMCs are subject to epigenetic modification like many other cardiac cell types. Often, these histone regulators or DNA modifiers influence the differentiation of the SMC (49, 50). Specifically, there is a crosstalk between myofibroblasts and SMCs during hypoxia related stress (51, 52). Many of the epigenetic regulatory pathways between fibroblasts and SMCs are not widely understood. However, DNA demethylation is one of the most well-known pathways in which epigenetic changes are carried out on molecules *in vivo* and *in vitro*. In a study by Watson et al.*,* reduction of DNMT3B levels in correlation to the expression of fibrosis-related genes was examined. It was found that CFs with lower levels of DNMT3B were associated with reduced expression of pro-fibrotic genes attributed to α-SMA and Collagen 1 (52). Specifically, α-SMA correlates with higher levels of DNMT3B and subsequently, with fibroblasts undergoing differentiation leading to pathological fibrosis. The epigenetic relationship of CFs and α-SMA can be further illustrated in a study by He et al.*,* in which CFs treated with TGF-β1 *in vitro* increased the expression of α-SMA through epigenetic modifications. In the same study, a connection was found between TGF-β1 inhibiting DNMT1 expression while cardiac fibroblasts were going through differentiation (53).

The specific pathways in which SMCs are upregulated are complex and poorly understood. In a study conducted by Yang et al.*,* myocardin related transcription factor A (MRTF-A) mediated the expression of proinflammatory genes through endothelin in vascular smooth muscle cells (VSMCs). MRFT-A was also found to promote an increase in histone modifications on genes after the addition of endothelin in cell culture. In silencing MRTF-A downstream effects of the expression of IL-6, MCP-1, and IL-1 were decreased through ET-1's (54). VSMCs varying expressions can affect the inflammatory response in the heart.

Interestingly, there have been connections between vascular stiffness associated with crosstalk of CFs and SMCs, specifically in aging mechanisms in the heart (55, 56). One study found that the mineralocorticoid receptors on SMCs decreased epigenetic regulation causing increased vascular stiffness. These receptors caused a decrease in EZH2 signaling, therefore suppressing H3K27me3 which was directly associated with this stiffness and vascular dysfunction in the heart (57). Another study found that the CFs related ECM dysfunction directly connected to the SMCs decline in density. The study connected these finding specifically to TGF-β signaling between the CFs and the SMCs (58).

In a study by Huo et al.*,* protein arginine methyltransferase 5 (PRMT5) was found to be upregulated in VSMCs that were treated with platelet-derived growth factors. Furthermore, the same study found that excessive amounts of PRMT5 suppressed SMC genes and switched to VSMC remodeling associated with injury. This pathway was discovered to be histone dimethylation of H3R8 and H4R3 after increased levels of PRMT5, which then triggered a decrease in the acetylation of H3K9 and H4, overall causing a change in SMC gene expression (59). This research shows a promising start to how other pathological conditions in the heart may be attributed to, and affected by, differing epigenetic modifications between CFs and SMCs.

### 2.3 Epigenetic regulation between cardiac fibroblasts and immune cells

The balance between immune cells and cardiac fibrosis is vital for cardiac repair mechanisms. Immune cells often work in phases that aid in wound healing after cardiac damage, in which macrophages, dendritic cells, mast cells, and T cells are of great importance at different times (60). It has been identified that cardiac fibrosis triggers an immune response (61). But further investigation is starting to highlight the role that epigenetic regulation plays on communication between these dynamic cell types. For example, HDACs have been shown to regulate fibrosis and fibroblasts through immune cell pathways involved in apoptosis, oxidative stress, and inflammation (62). A specific HDAC family, Sirtuin 3 (SIRT3), has been shown to regulate the crosstalk between fibrosis and inflammation. SIRT3 works by regulating the AP-1 pathway associated with oxidative stress. More directly, the loss SIRT3 regulation as a histone 3 lysine 27 deacetylase caused increased fibrosis and inflammation associated with an immune response (63). Epigenetic modifications to pathological CFs, also called myofibroblasts, have been shown to alter immune related responses via interleukin 1 beta (IL-1β). This study showed the communication due to IL-1β increased remodeling and was potential therapeutic for pulmonary arterial hypertension (64).

A study exploring the role of crosstalk between macrophage and CFs determined that these immune cells help with collagen deposition during scar formation (65). More importantly, there is evidence to suggest that epigenetic regulation strongly influences this macrophage mechanisms via histone modifications and transcriptions factors (66). The epigenetic landscape of macrophages also has been shown to regulate crosstalk between fibroblasts in atherosclerosis. It has been studied that DNA methylation and long non coding RNAs can epigenetically alter the function of macrophages and can dynamically alter inflammation around plaque formation (67). Additionally, Kuznetsova et al. describe lineage-determining transcription factors (LDTFs) and signal-dependent transcription factors (SDTFs) as major regulators the macrophage epigenetic landscape. It is described that these factors contribute to the development, identity, and memory of the immune cells, which therefore affects the response to atherosclerosis, scarring, and pathological fibrosis (67).

The role of long noncoding RNAs (lncRNAs) has been suggested as an epigenetic mechanism between fibroblasts and inflammation. Specifically, a study found that DNMTA3A methylation decreased the expression of a lncRNA named NEAT1. This caused and increased in a CF inflammatory form of cell death called pyroptosis (68). In addition, NEAT1 has been shown to drive cardiac fibrosis through recruiting EZH2 to the promoter region of SMAD7 to ultimately inhibit its function (69). SMAD7 along with SMAD6 are inhibitory SMADs that act to inhibit the downstream targets of the TGF-β signaling pathway which can reduce fibrotic gene expression (70). Knockdown or inhibition of NEAT1 has been shown to attenuate fibrosis remodeling while overexpression exacerbates it. Looking more into other mechanisms that involve NEAT1 will be important in designing therapies against cardiac dysfunction.

Another study found that epigenetic regulations of a few genes, such as NLRP3, MMP2, MMP9, CCN2/CTGF, and AGT played a major role in cardiac fibroblast regulation as well with monocytes and neutrophils. Many of the regulators were described as super enhancers of DNA hypomethylation that appeared to regulate this crosstalk between fibroblast and various immune cells (71).

### 2.4 Epigenetic regulation between cardiac fibroblasts and cardiac endothelial cells

Among the cells in the heart, cardiac endothelial cells (ECs) make up around 60% of the total nonmyocyte cell composition (25). In the heart, ECs are primarily responsible for regulating angiogenesis, tissue remodeling, neovascularization, bloody supply, and ECM composition (72). By understanding epigenetic mechanisms between CFs and ECs, we can target specific pathways, like angiogenesis, to better understand how the heart functions. For example, disruption of the intercellular connections between these two cell types within the heart results in cardiac pathological remodeling. It was revealed that SUMOylation (SUMO), a dynamic post-translational epigenetic mechanism, has a significant role in cardiac function after MI (73). Interestingly, the SUMO1 gene expression has different effects based on the cell type. In CFs and ECs, total deletion of the SUMO1 gene exacerbated the effects of myocardial injury, specifically in the fibroblasts to myofibroblast transition. Interestingly, the SUMO1 knockout (SUMO1-/-) was beneficial for promoting neovascularization processes in ECs after MI. Transdifferentiation from basal state CFs to terminal state myofibroblasts is an important step for cardiac repair after myocardial injury. SUMO1-/- also expanded the CF subset 4, an indicator of fibroblast transition into myofibroblasts (73).

The advancement of genomic technology in the past few decades has revolutionized the way scientists examine cell processes and signaling. A study by Jiang *et al*. utilized the epigenetic tool CRISPR-associated synergistic activation mediator (CRISPRa-SAM) system. This tool was capable of activating endogenous genes to induce reprogramming mechanisms in CFs. Genome editing through CRISPR has pioneered science and research to treat many different genetic-related diseases (74). Researchers have exploited CRISPR technology to pave the way for new therapeutic mechanisms such as gene regulation, epigenome modifications and chromatin manipulation to name a few (74). By using this tool, the scientists in this study were able to reprogram CFs into CRISPR-induced cardiovascular progenitor cells (ciCPCs) (75). Once in this state they had the ability to further remodel the ciCPCs into ECs. The generated ECs contributed to neovascularization and increased bloody supply in the murine hearts (75). With this discovery we can incorporate the fibroblast-derived ciCPCs into diseased hearts to ensure adverse cardiac remodeling effects are limited.

As mentioned before epigenetic regulation through histone modifications significantly impacts gene transcription. The removal of chemical residues on the histone tails modifies the chromatin structure thereby causing gene repression. Recently, researchers discovered a novel role of lysine-specific demethylase 5B (KDM5B) in cardiac remodeling. It has been shown in previous studies that abnormal regulation of KDM5B can lead to a multitude of disorders including oncogenesis and immune system dysfunction (76, 77). By knocking out KDM5B expression in mice, scientists were able to study the changes in CFs and ECs (78). After induced MI, the mice showed minimal mRNA and protein expression of KDM5B as well as no expression in the nuclei of KDM5B-deficient CFs. TGF-β stimulation on KDM5B-KO mice suppressed profibrotic genes involved in collagen fibril and ECM organization. Additionally, control wild-type mice were treated with GSK467 (an inhibitor of KDM5B), which repressed profibrotic genes and decreased the production of α-SMA, an indicator of fibroblast differentiation to myofibroblast (78). Worth noting is the ability of CFs to take on an endothelial cell state by knocking out KDM5B. Increased expression of positive angiogenesis-related genes, such as Vascular endothelial growth factor A, Fibroblast growth factor 1, and Protein kinase C alpha, and decreased expression of negative angiogenesis-related genes, such as C-C chemokine receptor 2, Sulfatase 1, and Thrombospondin 2, were found during the screening of KDM5B-KO mice (78). KDM5B deficiency also enhanced endothelial marker genes and tube formation fortifying angiogenesis in the myocardium after MI.

Expanding on histone modifications, Class 2A HDACs are associated with gene silencing and play an important role in regulating angiogenesis within ECs. HDAC9 has been shown to actively repress the transcription of miR-17-92 which promotes angiogenesis, vascular growth, and cardiac remodeling in ECs of mice & zebrafish (79). The silencing of this histone modifier exacerbated the effects of miR-17-92 induce antiangiogenic properties within these *in vitro* and *in vivo*.

Endothelial-to-mesenchymal transition (EndMT) is an important process in cardiovascular development and pathological conditions. Here endothelial cells have the ability to undergo a morphological change into a fibroblast by repressing endothelial genes and upregulating fibrotic and ECM related genes (80). During damage and repair EndMT contributes to a significant portion of the amount of CFs in the heart (81). This cell fate can be driven by TGF-β and when dysregulated can lead to pathological conditions such as atherosclerosis and cardiac fibrosis (81, 82). Researchers have been searching for ways in which the EndMT process can be regulated to protect the heart during development and through disease. One study points to a lncRNA, metastasis-associated lung adenocarcinoma transcript 1(MALAT1), which can modulate TGF-β-induced EndMT by regulating miR-145 and the genes of TGF-β receptor 2 and SMAD3 (83). Interestingly, miR-145 and MALAT1 bind reciprocally to each other allowing for co-regulation. Another study highlights BRD4 as a notable epigenetic reader protein that is highly expressed in ECs which contributes to EndMT and cardiac fibrosis (84). As mentioned before, BRD4 can be inhibited by JQ1 which can help preserve EC cell sprouting and attenuate pathological conditions.

Expanding on the influence of lncRNAs, the silencing of lncRNA maternally expressed gene 3 (MEG3) serves as a cardioprotective mechanism by regulating ECM gene expression and limiting fibrosis and hypertrophic remodeling (85). MEG3 is highly enriched in murine CFs after pressure overload. ECs also express this lncRNA, although, at a much lower level. MEG3 has been reported to have antiangiogenic properties, and its knockdown prevents the induction of MMP-2 by regulating the transcriptional activity of tumor protein 53 on the promoter of MMP-2.

Therefore, by understanding the epigenetic crosstalk between CFs and ECs we can identify targets that induce profibrotic behaviors that lead to CVDs. Among the epigenetic mechanisms mentioned between these two cell types, there are many more that are actively being discovered. When they arise, it will help in the development of treatments for pathological cellular crosstalk in the heart. Also, the use of the CRISPR system and similar technology will continue to uncover new apparatuses that will help in cardiac regeneration.

## 3 Discussion

Epigenetic modifications have attracted great interest over the recent decades allowing for the expansion of our knowledge within the health and diseased states of the heart. Coupled with the current understanding of intercellular communication mechanisms, we can foresee profound advancements to cardiovascular therapies. The significance of epigenetic regulation of CFs has been indispensable in a variety of biological activities. Since all cardiac cell types are in contact with CFs or the ECM, it is important that we inspect the crosstalk between these environments and their downstream effects, especially in times of injury or stress in the heart. Here we summarize the vital role of epigenetic modifiers such as DMNTs, miRNAs, lncRNAs, histone modifiers as well as other molecules that package and transport these regulators between CFs and other cardiac cells. Additionally, paracrine transmission mechanisms, transcription factors and post-translational modifiers that mediate cell signaling, fate, identity and function, were discussed.

Despite this comprehensive review, it is important to note that epigenetic mechanisms within the heart interact with many other biological molecules such as metabolites, carcinogens, and senescence agents which remain to be fully elucidated. Similarly, the efficacy of treating neonatal cell types can be difficult in predicting errors in molecular mechanisms since many heart diseases develop later in adulthood. Future breakthroughs within the field of epigenetics will provide new treatments to either slow the progression or reverse the effects of CVD.

With the development of high-throughput and single-cell sequencing analyses in recent years it is probable that we will soon be able to understand many more epigenetic modifications that take place in CFs. We have provided insight into the use of CRISPR-Cas9 as another staple technological tool to aid in the reprogramming of CFs into ECs to promote angiogenesis. Similarly, another natural reprogramming process called EndMT has been highlighted to show the importance of maintaining cardiac homeostasis. Nevertheless, the causation and molecular process of EndMT is merely just beginning to be expounded. Interestingly, a recent study has provided a source of non-canonical EndMT signaling called transient fibrotic-like phenotype (EndoFP) in response to TAC-induced pressure overload conditions. These ECs had a transient expression of fibrotic genes and an oddly low expression level of α-SMA (86). Additionally, when co-cultured with CFs and CMs, fibrotic and hypertrophic genes were noticeably upregulated. It was concluded that insulin-like growth factor-binding protein 5 mediated the crosstalk between these cell types presenting a new therapeutic approach for pressure overload. Another related process heavily studied is the epithelial-to-mesenchymal transition in which epicardial cells can differentiate into fibroblasts, smooth muscle cells, or endothelial cells to develop the vasculature of the heart (87, 88).

Other avenues of therapeutic intervention include mediating paracrine/cytokine signaling processes. In agreement with other current studies, it is prevalent that canonical signaling pathways such as Ang II and TGF-β drive cardiac dysfunction by activating profibrotic and proinflammatory genes (89, 90). Although TGF-β can promote unfavorable effects within the heart, is it essential for maintaining homeostasis of the cardiac milieu. We have highlighted some epigenetic factors that activate or co-regulate the crosstalk between CFs and other cardiac cell types through such signaling. Further studies, should explore the role of cardiac cell crosstalk and epigenetic modifications with aging mechanisms. With changes in cardiac cell phenotype over time, it would be important to introduce the additional variable of age to crosstalk in the heart. With CVD being a disease that often affects older populations, understanding the changes in epigenetic regulation of crosstalk in the heart over time would be extremely important for understanding the realities of this devastating disease.

Whether under normal conditions or in response to stress stimuli, intercellular communication is always present in the development of the heart, and throughout our lives. Given the important role CFs play in heart function, it is imperative to consider the implications of epigenetic modifications. This review has concisely highlighted and compounded the recent evidence that supports the epigenetic mechanisms that govern the crosstalk between CFs and multiple other cardiac cell types. As our understanding of epigenetic regulation in cardiac cell crosstalk increases, we can develop novel therapeutic mechanisms to mitigate the risk and development of CVDs.

## 4 Conclusions

There are multiple variations in the epigenetic modifications that regulate crosstalk between cardiac fibroblasts and other cardiac cell types. Despite the growing bodies of literature on the crosstalk between cardiac fibroblasts and other vital cell types within the heart, there is still more research that needs to be done on the direct epigenetic modifiers, small RNA molecules, and signal proteins that lead to a pathological state in the heart. With deaths attributed to cardiovascular disease continuing to be one of the leading causes of death each year, it is crucial that more research is dedicated to these mechanisms, more specifically how we may be able to reverse them in patients who suffer from decreased cardiac function.
